# The Relationship between Heart Rate Variability, Pain Intensity, Pain Catastrophizing, Disability, Quality of Life and Range of Cervical Motion in Patients with Chronic Non-Specific Neck Pain: A Cross-Sectional Study

**DOI:** 10.3390/healthcare12111055

**Published:** 2024-05-22

**Authors:** Ioannis Kyrosis, Eleftherios Paraskevopoulos, George A. Koumantakis, Anna Christakou

**Affiliations:** 1 Department of Physiotherapy, University of Peloponnese, 23100 Sparta, Greece; pth19066@uop.gr; 2 Laboratory of Advanced Physiotherapy, Department of Physiotherapy, University of West Attica, 12243 Athens, Greece; elparaskevop@uniwa.gr (E.P.); gkoumantakis@uniwa.gr (G.A.K.); 3 Laboratory of Biomechanics, Department of Physiotherapy, University of Peloponnese, 23100 Sparta, Greece

**Keywords:** heart rate variability, chronic neck pain, pain catastrophizing, cervical range of motion, quality of life

## Abstract

The purpose of the present cross-sectional study was to examine the relationship between heart rate variability (HRV) and the range of cervical motion, disability, pain intensity, pain catastrophizing, and quality of life in patients with chronic, non-specific neck pain. Thirty-five patients, aged 20–48 years, with chronic non-specific neck pain, completed validated questionnaires regarding neck pain intensity, pain-associated disability, catastrophic thoughts, and quality of life. The range of cervical motion was assessed using a digital goniometer. HRV indices were recorded in three positions (supine, sitting, and standing) through a smartphone application. Several significant correlations were observed between HRV indices and neck pain disability, the helplessness factor of catastrophizing, neck rotation, and quality of life. These correlations were only observed in the standing position. Pain catastrophizing was positively correlated with disability and pain intensity during active neck movement (Pearson r = 0.544, *p* < 0.01; Pearson r = 0.605, *p* < 0.01, respectively). Quality of life was negatively correlated with pain intensity during active movement (Pearson r = −0.347, *p* < 0.05). HRV indices were correlated with the psychological and physical domains of neck pain. These cardiac indices have been related to neck pain variables in some previous studies. Further research is needed to confirm this relationship in different daily conditions.

## 1. Introduction

Between 50% and 85% of the general population experience neck pain at some point in their lives [1]. Chronic neck pain is a musculoskeletal disorder characterized by continuous or recurrent pain in the cervical spine region lasting for at least three months [2]. Chronic neck pain can lead to a significant decrease in the range of cervical motion and neck muscle strength. The 12-month prevalence of neck pain typically ranges between 30% and 50%; the 12-month prevalence of activity-limiting pain is 1.7% to 11.5% [3]. Patients with chronic neck pain often experience psychological disorders like anxiety or depression [4], as well as sleep problems [5], which can negatively affect their quality of life.

The relationship between the Autonomic Nervous System (ANS) and the pain control system is well-established. This connection becomes increasingly evident due to the overlap between cerebral structures responsible for regulating the ANS and those involved in pain modulation [6]. Heart Rate Variability (HRV) involves the measurement of fluctuations in heartbeats across successive cardiac cycles, providing an estimation of an individual’s ANS activity [7]. The Neurovisceral Integration Model [6] posits that increased Heart Rate Variability (HRV) enables individuals to adapt to internal and environmental changes through a ‘central autonomic network’ [8].

Some studies have assessed the ANS behavior over the cardiovascular system in chronic pain patients, especially patients with low back pain [9]. HRV has been used as an evaluation method for the ANS function. ANS dysfunction has been correlated with chronic pain without the establishment of a clear causal relationship between them [9,10,11]. Increased sympathetic activity may play a role in the maintenance of neck pain symptoms [12]. Also, parasympathetic activation is involved in the inhibition of inflammatory processes [13]. Resting HRV is negatively associated with systemic levels of pro-inflammatory cytokines [14,15]. Some studies show higher concentrations of pro-inflammatory cytokines among patients with work-related neck pain [16]. In chronic muscle pain conditions, sympathetic activity due to nociceptive stimulation may cause disturbances in blood flow regulation in the affected muscle and enhance or prolong muscle activation. Mental stress may negatively affect this situation [17]. Shiro et al. [18] demonstrated that insufficient muscle blood flow and oxygenation of the trapezius muscles might be a result of the lower sympathetic nerve response during isometric exercise in patients with neck and shoulder pain compared with asymptomatic individuals [18]. The enhanced and prolonged muscle activation, due to disturbed blood flow and oxygenation regulation resulting from elevated sympathetic arousal, may then be associated with restricted cervical range of motion. This model could explain the association between HRV, which reflects sympathetic and parasympathetic activity, and cervical range of motion. Enhanced parasympathetic activity is associated with higher HRV indices [19]. Epidemiological studies indicate a positive correlation between increased physical activity during leisure time and improved vagal nervous system function [20,21].

There is limited research investigating the relationship between HRV and chronic neck pain [22,23,24,25]. Hence, the assessment of HRV in chronic neck pain warrants greater research attention. HRV could serve as an objective tool for characterizing chronic neck pain, and improvements in neck pain symptoms could be evaluated through changes in HRV indices [22,23,24,25]. Kang et al. [22] believe that the diagnosis of disability, through valid and reliable tools, such as the Neck Disability Index (NDI), and its relation to HRV may provide useful information on characterizing the severity of disability and recognizing factors responsible for disability in chronic neck pain patients [22]. A better understanding of the relationship between HRV and chronic neck pain may facilitate the development of novel therapeutic approaches that target the ANS and, consequently, alleviate chronic neck pain.

The psychological variables of neck pain and their relationship with HRV have not been thoroughly investigated to date. Similarly, there is a reported lack of studies on the connection between cervical spine range of motion and HRV. Moderate correlations have been found between pain intensity and neck kinematic parameters [26]. A study conducted by Chiu et al. [27] suggested correlating pain intensity with psychological variables and active range of motion, a methodology that could be easily incorporated into research on chronic non-specific neck pain [27]. This is particularly pertinent given the lack of established relationships between emotional states and self-reported pain or disability in chronic, non-specific neck pain [28].

Therefore, the aim of the present study was to assess the relationship between HRV, pain intensity, pain catastrophizing, disability, quality of life, and range of cervical motion in patients with chronic, non-specific neck pain. The first hypothesis posited that HRV would exhibit negative correlations with neck pain intensity and neck pain disability. The second hypothesis suggested negative correlations between range of cervical motion and quality of life with chronic neck pain, while catastrophizing would be positively associated with chronic neck pain.

## 2. Materials and Methods

### 2.1. Participants

The present study is a cross-sectional study. Thirty-five patients with chronic, non-specific neck pain participated in the present study. The participants were recruited from private physiotherapy clinics, and they had previously visited an orthopedic specialist where their neck region had been examined with an MRI.

The inclusion criteria for the sample were as follows: (a) aged between 18 and 48 years, regardless of sex; (b) diagnosed with chronic non-specific and non-radiating neck pain (>90 days) [29]; and (c) not engaged in regular training over the past 12 months, namely not undertaken >150 min of moderate-intensity, or >75 min of vigorous-intensity aerobic physical activity, or some equivalent combination of moderate-intensity and vigorous-intensity aerobic physical activity, per week. Moderate-intensity physical activity and vigorous-intensity physical activity, on a scale relative to an individual’s personal capacity, are usually described as a 5 or 6, and a 7 or 8, respectively, on a rating scale of perceived exertion of 0–10 [25,30]. Neck pain was defined as a score of ≥5 on the Neck Disability Index (NDI) and a score of ≥3 on the Numerical Rating Scale (NRS) at rest or during active cervical movement [25,31].

Exclusion criteria for the sample included: (a) history of neck injury and other pathologies of the cervical spine such as radiculopathy; (b) surgery in the head, face, cervical spine, upper or lower limbs, or cervical hernia; (c) degenerative disease of the spine; (d) physiotherapy treatment within the past 3 months; (e) use of analgesic, anti-inflammatory, or muscle relaxant drugs within the previous week; and (f) presence of any rheumatological or cardiovascular disease, chronic neurological or psychiatric disorders, drug addiction, anemia, or diabetes.

Participants provided written, informed consent and performed according to the Declaration of Helsinki. The study was approved by the ethics committee at the University (Number: 12097/27-11-2023).

### 2.2. Measurements

*Numerical Rating Scale (NRS) [32]*

The NRS is a scale with a score from 0 to 10, where 0 means “no pain” and 10 means “the worst pain you can imagine”. It is a valid and reliable scale [32]. The maximum pain during the last 24 h was recorded for each subject.

*Neck Disability Index (NDI) [33]*

NDI is the most widely used self-reported questionnaire for neck pain-disability [34]. It consists of 10 questions related to neck pain and neck pain-disability. Each question includes 6 possible answers, ranging from 0 to 5 points. A total score of 0–4 represents no disability; 5–14 mild disability; 15–24 moderate disability; 25–34 severe disability, while 35–50 equates to a complete disability. NDI has been validated for the Greek population [35]. Previous studies have shown high test–retest reliability (0.89, *p* < 0.05) and internal consistency (Cronbach α = 0.80) [33,36].

*Pain Catastrophizing Scale (PCS) [37]*

It is a valid and reliable Likert-type self-reported scale for pain-related catastrophizing that consists of 13 questions examining 3 factors. These factors are: (1) Rumination, (2) Magnification, and (3) Helplessness. Each question’s score ranges from 0 (no negative thoughts or feelings) to 4 (worst negative thoughts or feelings), and the total score ranges from 0 to 52 points. A higher score means increased levels of pain that are catastrophizing. PCS has been translated and validated for the Greek population [38]. Also, it has been validated for the Greek population with neck pain, indicating very good internal validity and internal consistency (Cronbach α = 0.95), as well as high test–retest reliability (ICC = 0.85) [39,40].

*EuroQol (EQ-5D-5L) [41]*

It is a standardized generic measure of health status. This instrument depends on a descriptive system that assesses health in five dimensions: mobility, self-care, usual activities (e.g., work, study, housework, and family or leisure activities), pain/discomfort, anxiety/depression. Each dimension is characterized by five levels of severity (no problems, slight problems, moderate problems, severe problems, extreme problems/unable to). The second part of the questionnaire consists of a visual analog scale (VAS), ranging from 0 to 100, which provides the respondent’s overall current health status. The EQ-5D-5L is commonly employed in clinical and economic evaluations of healthcare interventions due to its high reliability, responsiveness, and strong validity. Moreover, it requires only a few minutes to complete [42]. It has been translated and validated in the Greek language [43].

*Goniometry*

Digital goniometry measures the cervical range of motion (flexion, extension, rotation, side flexion). Goniometry measurements have been shown to be reliable (ICC between 0.79 and 0.97) with a small error of measurement (between 1.40° and 3.35° for all movements) [44]. In the present study, a valid and reliable digital goniometer (Digital Angle Ruler 200mm, TOTAL One-Stop Tools Station) was utilized, providing measurement accuracy up to two decimal places.

*HRV4Training Application*

It is a popular smartphone application being used mostly by athletes. It measures different HRV indices through photoplethysmography (PPG). Participants can use the HRV4Training application by placing their index finger on the phone camera, which measures the amount of light absorbed and reflected by the finger. The phone-based PPG has been validated and confirmed to provide reliable HRV recordings [45,46]. HRV4Training application [47] serves as a fast and valid alternative to the electrocardiogram (ECG), the gold-standard method of measurement in research [19,48,49]. Mobile phone PPG through HRV4Training had an almost perfect correlation with the ECGs (r = 0.99) [50]. Mobile phone cameras have a low frame rate, necessitating the use of different signal-processing techniques to capture HRV measurements from the phone video stream [45]. The HRV4Training app up-samples the signal between 30 and 180 Hz [50]. HRV4Training implements a peak detection algorithm to determine peak-to-peak intervals from up-sampled PPG data. Peak detection is based on a slope inversion algorithm [48]. In this study, an Honor 8A smart phone was used [(model JAT-L29; main camera: 13 MP, f/1.8, PDAF; OS: Android 9.0, EMUI 9; CPU: Octa-core (4 × 2.3 GHz Cortex-A53 and 4 × 1.8 GHz Cortex-A53)]. The HRV4Training application detects right away if your phone is supported [38]. Our phone was compatible with the app (it costs 11, 99 €).

### 2.3. Procedure

**Figure 1:**
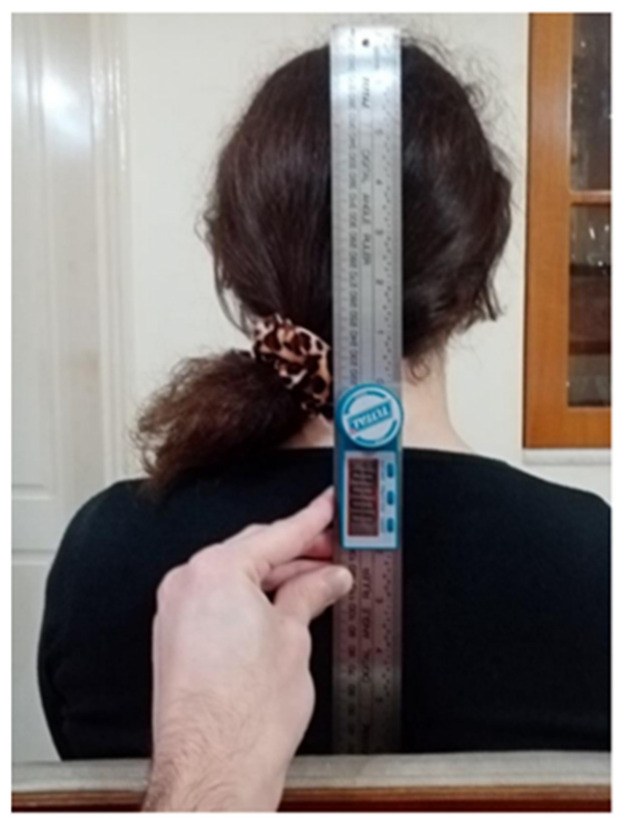
Starting position of right cervical side flexion measurement.

**Figure 2:**
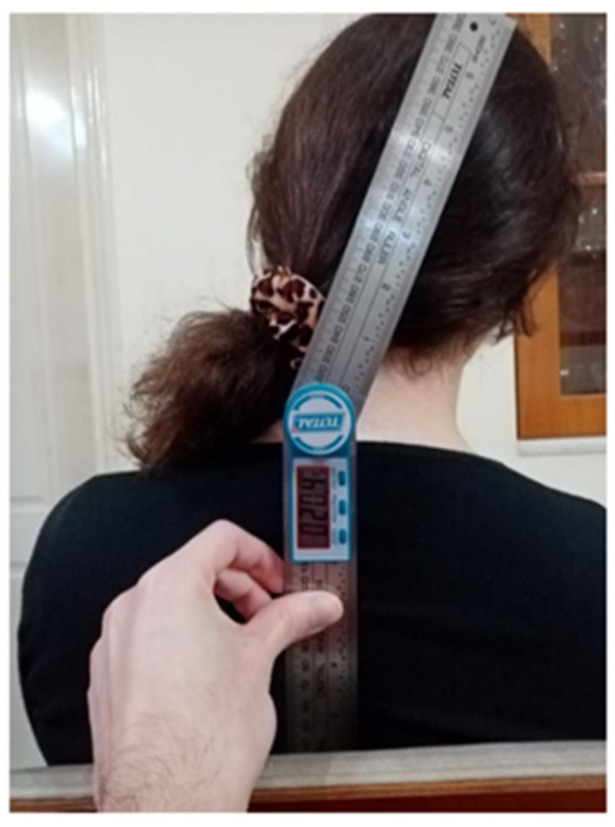
Ending position of right cervical side flexion measurement.

The participants were recruited by word-of-mouth in the local physiotherapy clinics. All the measurements were carried out by the last author in the local physiotherapy clinics. Firstly, participants assessed their pain intensity in a resting condition (NRSr) and after they performed one repetition of active cervical spine movements of flexion, extension, rotation, and side flexion (NRSm). The highest level of pain experienced during any of these movements was recorded. Participants were also questioned about the last time they felt neck pain. After pain evaluation through NRS, participants completed the NDI for self-assessment of neck pain-associated disability, PCS for pain catastrophizing, and EuroQol for general physical, mental health, and quality of life. After completing all the questionnaires, patients underwent cervical range of motion measurements using the digital goniometer. Specifically, flexion, extension, right and left lateral flexion, and right and left rotation of the neck were measured. Patients were seated in a chair with their backs supported, their feet on the ground, knees and ankles bended in 90°, and their hands on their thighs. Each neck movement was performed 3 times in a pain-free range with a normal speed, and the best result was recorded. They were instructed to move only their head and not their shoulders during the measurement (Figure 1 and Figure 2).

**Figure 3:**
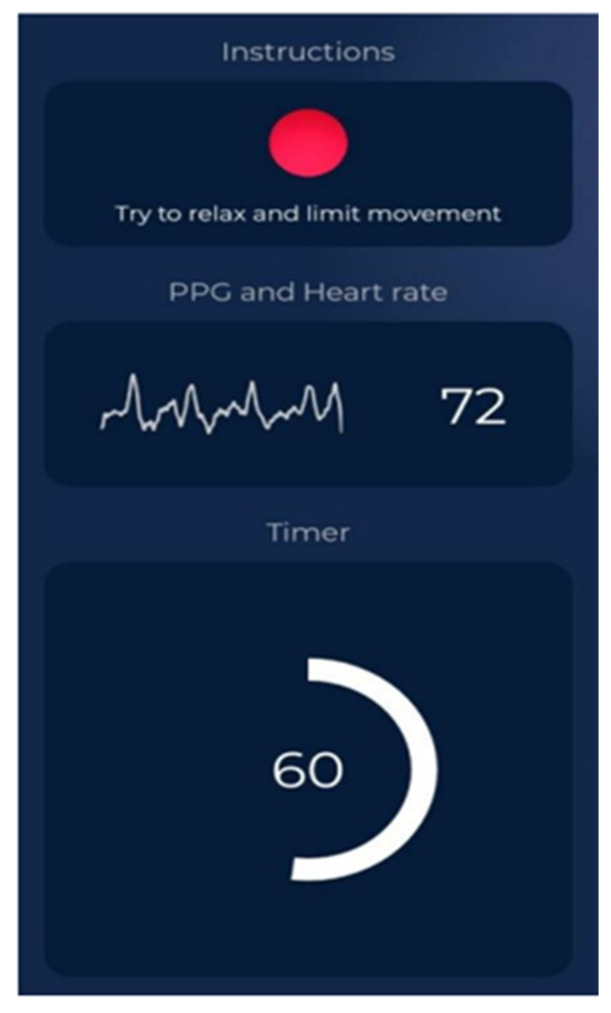
HRV measurement through HRV4Training application.

**Figure 4:**
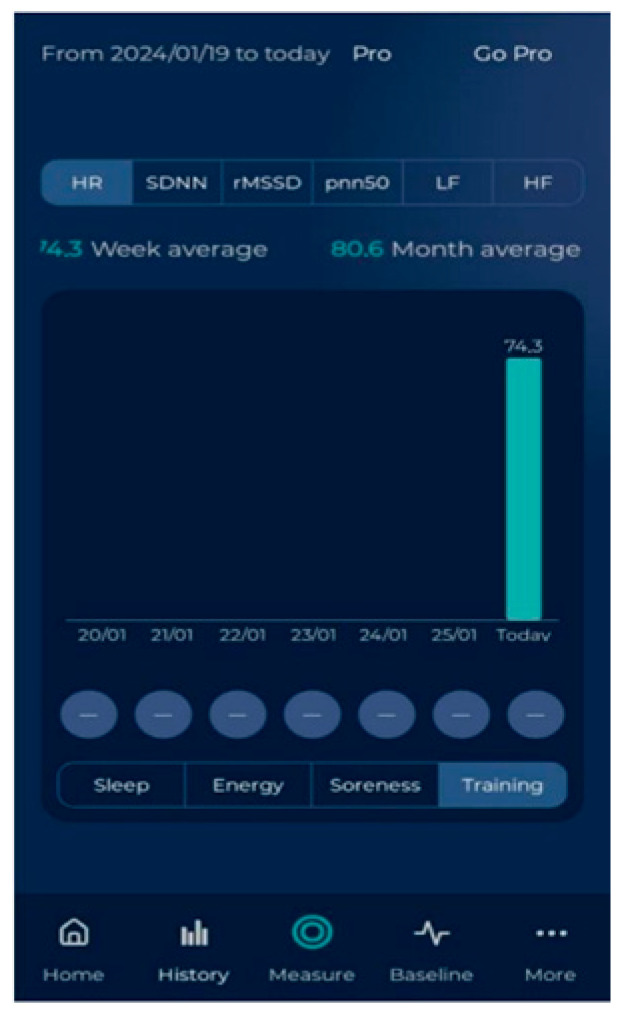
Heart Rate indication of a patient through HRV4Training application.

Then, HRV measurements were recorded with the HRV4Training application (Figure 3). All participants had been instructed not to consume caffeinated or alcoholic beverages 12 h before the testing procedure. Before data collection, each participant rested in a supine position for 10 min to ensure Heart Rate (HR) stabilization. HRV was then recorded from the supine, sitting, and standing positions, with a 2 min recording for each position and a 1 min rest between position change (8 min duration in total). Participants were told not to talk during HRV recording. They were also instructed to relax and breathe in a normal way and to stay as stable as possible. They used their right index finger due to the camera’s placement on the right side of the mobile phone. Data were automatically analyzed by the application, which emitted noise in cases of poor measurement quality. Measurements were repeated until optimal quality was achieved. Time-domain and frequency-domain HRV analyses were performed at rest using widely accepted indices [SDNN, Standard Deviation of Normal—to—Normal RR intervals; RMSSD, Root Mean Square of the Successive Differences; pNN50, NN50 count (Number of pairs of adjacent NN intervals differing by more than 50 ms in the entire recording) divided by the total number of all NN intervals; LF, Low Frequency; HF, High Frequency] [51]. HR values were also obtained (Figure 4).

### 2.4. Statistical Analysis

Descriptive statistics were used to examine the demographic data of the sample. A normality test of the distribution of all variables was conducted using the Kolmogorov–Smirnov test.

Pearson’s r correlation coefficient analysis was performed on the following variables: HRV, Heart Rate (HR), SDNN, RMSSD, LF, and LF/HF from the supine position; HRV, HR, SDNN, and LF/HF from the sitting position; HRV, HR, RMSSD, LF, and HF from the standing position; as well as the Numerical Rating Scale for neck pain intensity (NRSm), Neck Disability Index (NDI), Pain Catastrophizing Scale (PCS), Rumination, Helplessness, EQ-VAS (Visual Analog Scale for quality of life), and range of motion measurements (Flexion, Extension, Left Rotation) of the neck.

Spearman’s r correlation coefficient analysis was used to assess the relationship between the following variables: pNN50 and HF from the supine position; RMSSD, pNN50, LF, and HF from the sitting position; SDNN, pNN50, and LF/HF from the standing position; as well as chronicity of pain, the Numerical Rating Scale for neck pain intensity (NRSr), Magnification, the descriptive system of EuroQol, and range of motion measurements (Right lateral flexion, Left lateral flexion, Right rotation) of the neck. Data were analyzed using the IBM Statistical Package for the Social Sciences (SPSS) 23.00 with a significance level α = 0.05 and a stricter significance level α = 0.01 to avoid the risk of having Type I error.

## 3. Results

The demographic characteristics of the sample, as well as pain and HRV measurements, are presented in Table 1. Pearson’s r correlations between HRV indices, pain questionnaires, and range of cervical motion are shown in Table 2. The results revealed significant correlations among participants in the standing position, including HRV with the Neck Disability Index (NDI) (r = −0.408, *p* = 0.015), HRV with the helplessness factor of the Pain Catastrophizing Scale (PCS) (r = −0.370, *p* = 0.029), HRV with left rotation of the neck (r = 0.346, *p* = 0.042), RMSSD with NDI (r = −0.385, *p* = 0.022), and RMSSD with helplessness (r = −0.371, *p* = 0.028).

Table 3 displays Spearman’s r correlations between HRV indices, pain questionnaires, and range of cervical motion. Significant correlations were observed between the standing position, including pNN50, with the score of the descriptive system of EuroQol (r = 0.384, *p* = 0.025), pNN50 with right neck rotation (r = 0.450, *p* = 0.007), LF/HF and the score of the descriptive system of EuroQol (r = −0.393, *p* = 0.019), and LF/HF with right neck rotation (r = −0.352, *p* = 0.038).

Additional significant correlations between pain variables are presented in Table 2 using Pearson’s r correlation coefficient and in Table 3 using Spearman’s r correlation coefficient.

If the significance level was set at α = 0.01, then the only significant correlation with an HRV index would be observed from the standing position of pNN50 with the score of the right neck rotation (r = 0.450, *p* = 0.007). Also, statistical correlations between NRSr and chronicity (r = 0.549, *p* = < 0.001), LLF and RLF r = 0.603, *p* = < 0.001), RR and EuroQol (r = 0.516, *p* = 0.001) were found. Additional significant correlations between pain variables are presented in Table 2 using Pearson’s r correlation coefficient.

## 4. Discussion

The present study did not find a relationship between pain intensity in resting conditions or during active cervical spine movement and HRV indices, contrary to the study hypothesis. A similar finding was observed in patients with chronic low back pain, where reduced HRV was significantly correlated with higher perceived disability but not with pain intensity itself [10]. It is possible that the measurement duration of HRV in the present study was not sufficient to detect a correlation between pain intensity and cardiac indices, especially considering that some patients did not experience neck pain during measurement.

Moreover, this study did not find any relationship between neck ROM and pain intensity, which aligns with similar findings reported by Chiu et al. [27]. In their study, they found a weak association between pain and active cervical motion before intervention on the impact of a 6-week physiotherapy treatment on individuals with chronic neck pain. Interestingly, correlations tended to increase in follow-up assessments conducted at 6 weeks and 6 months after the initial assessment. They suggested that physical impairments represent only one aspect of a clinical problem and advocated for future studies to explore the relationship between pain intensity and psychological parameters such as kinesiophobia, as well as range of motion [27]. In the present study, while active cervical range of motion did not correlate with pain intensity, left rotation of the neck was negatively correlated with catastrophizing and rumination.

In line with the study hypothesis, pain catastrophizing was found to be significantly correlated with pain intensity during active movement, which is consistent with previous studies [52,53]. Dimitriadis et al. [28] investigated 45 patients with idiopathic chronic neck pain and found that anxiety was the only psychological condition significantly correlated with the intensity of neck pain. They also observed that pain-associated disability was more strongly correlated with catastrophizing than with pain intensity [28]. However, in the present study, disability showed a similar degree of correlation with catastrophizing and pain intensity during active neck movement. This finding may help explain the observed correlation between pain catastrophizing and pain intensity in our study.

In addition, quality of life was found to be negatively correlated with pain intensity during movement. Notably, in the present study, neck pain intensity was moderate both at rest and during movement. This moderate level of pain intensity may explain the fair degree of relationship between the EQ-VAS and NRSm. However, it is noteworthy that there was no correlation observed between pain intensity and the descriptive system of EQ-5D-5L.

Neck pain-associated disability was found to be negatively correlated with HRV and the vagal index RMSSD, but this association was observed only in the standing position. On the contrary, Santos-de-Araújo et al. [25] reported correlations between neck disability and HRV indices only in the supine and sitting positions. Additionally, Henley et al. [54] observed that a 50-degree head-up tilt resulted in sympathetic nervous system predominance, as evidenced by an increase in the LF/HF ratio [54]. These findings underscore the need for future studies to evaluate the effect of position on HRV measurement in individuals with chronic neck pain.

In the present study, quality of life was found to be positively correlated with the parasympathetic index pNN50 and negatively correlated with the LF/HF ratio in the standing position. This aligns with findings from Hallman et al. [55], who identified associations between some SF-36 sub-indices and HRV indices in patients with chronic neck pain. Specifically, they observed a positive correlation between social function and resting SDNN, as well as between emotional role and resting LF. Conversely, a negative correlation was detected between bodily pain and resting diastolic blood pressure [55].

The relationship between sociality and parasympathetic activity, as measured by SDNN, is consistent with theoretical predictions. Social interventions and compassionate relationships have the potential to influence the vagal nerve, which regulates visceral parasympathetic activity. Therefore, increasing parasympathetic tone, as indicated by HRV, could be considered an aim of modern psychotherapeutic interventions and serve as an outcome measure for comprehensive treatment [56].

Regarding any differences between the genders of the participants, Kang et al. [22] divided the entire sample into three groups (group 1: young participants, less severe symptoms, sex ratio similar to the original population; group 2: middle-to-older-aged women, the highest disability and the highest pain intensity; group 3: only male participants, lower disability levels and pain intensity than the women in group 2). They found that subjective disability was significantly correlated with HRV parameters. This correlation might be more obvious in women who had the highest disability scores [22]. This result confirms that chronic neck pain is more frequent in women [57]. More research should be conducted to confirm these gender differences.

The present study uniquely investigated the relationship between range of neck motion and HRV, uncovering correlations between these two variables. PNN50 from the standing position was positively correlated with neck right rotation, while the LF/HF ratio from the standing position was negatively correlated with right rotation. Generally, a higher vagal tone (higher HRV) leads to an appropriate behavioral adjustment and self-regulation [6]. Also, joint range of motion can be considered a behavioral parameter; therefore, an improvement in HRV levels may increase range of motion [58]. If the significance level was stricter, the only significant correlation with an HRV index would be observed from the standing position of pNN50 with the score of right neck rotation; thus, future research should aim to confirm this relationship.

### Limitations of the Study and Future Considerations

The present study findings should be interpreted in light of some limitations. Firstly, the sample size was small; the participants were physically inactive, with no homogeneity in terms of age, anthropometrics, or sex, with conservative care and probably limited alteration in ANS function due to mild neck pain. Therefore, the results of this study cannot be generalized to other clinical populations. Secondly, there are no normative values for accurately recording the time of each cardiac index. While the majority of studies support a 1 min recording as sufficient, the ultra-short-term (UST) HRV measurement (2 min) used in this study may have increased the validity of the findings compared to the 1 min measurement. Additionally, UST HRV measurement serves as a proxy for short-term (5-min) HRV measurement, which in turn is a proxy for long-term (24 h) HRV measurement, the gold-standard HRV recording period. Although UST measurement is a limitation of the present study, it can be considered a practical method for patients, or even the healthy population, to receive important health status information quickly. Clinicians could also gather useful patient characteristics. Thirdly, while ECG provides the most reliable HRV measurement, a smartphone app was used instead. Similar applications can easily be used on a daily basis due to the widespread possession of smartphones currently. Fourthly, a respiratory rate < 0.15 Hz affects HRV in the low-frequency spectrum, so assessing the respiratory rate of volunteers could facilitate HRV analysis, but it was not assessed in the present study. Finally, patients with chronic neck pain could experience decreased HRV before pain develops.

Future research with a larger and more homogeneous sample, long-term follow-up, and control group is warranted to accurately determine the relation between HRV indices and chronic neck pain in different populations. Moreover, including non-linear HRV indices to assess chronic neck pain at rest, during activities of daily living, or during training can provide a valid description of the autonomic nervous system (ANS) function. The present study can be extended to different categories of chronic neck pain, such as traumatic pain or neck pain with neurological symptoms.

## 5. Conclusions

This study identified correlations between linear HRV indices and various aspects of chronic non-specific neck pain, including pain intensity, range of cervical motion, pain catastrophizing, disability, and quality of life. Additionally, significant correlations between cardiac indices and pain variables were observed in the standing position. Increased rates of disability and catastrophizing, as well as lower levels of quality of life or decreased range of motion in these patients, were associated with decreased parasympathetic activity. Conversely, dominance of sympathetic activity was correlated with decreased quality of life and range of motion. Future longitudinal studies involving a larger number of chronic neck pain patients should be conducted.

## Figures and Tables

**Table 1:** Anthropometric and demographic characteristics, pain, and HRV measurements of the patients.

Characteristics		Mean ± SD
Age (years) ^a^		39.66 (8.49)
Height (m) ^a^		1.65 (0.07)
Mass (kg) ^a^		71.11 (11.22)
BMI (kg/m^2^) ^a^		26.01 (4.50)
NRSr		4.14 (1.73)
NRSm		5.11 (1.95)
NDI		11.77 (5.30)
HRV—Supine		6.78 (0.76)
HR (bpm)—Supine		73.55 (11.81)
SDNN (ms)—Supine		41.25 (13.88)
RMSSD (ms)—Supine		35.66 (16.06)
pNN50 (%)—Supine		15.23 (14.50)
LF (Hz)—Supine		0.05817 (0.02985)
HF (Hz)—Supine		0.06143 (0.02739)
LF/HF—Supine		0.96063 (0.32071)
HRV—Sitting		7.06 (0.58)
HR (bpm)—Sitting		76.26 (10.38)
SDNN (ms)—Sitting		50.89 (13.58)
RMSSD (ms)—Sitting		40.11 (13.71)
pNN50 (%)—Sitting		18.20 (13.51)
LF (Hz)—Sitting		0.07632 (0.044)
HF (Hz)—Sitting		0.08236 (0.042)
LF/HF—Sitting		0.97048 (0.365)
HRV—Standing		6.71 (0.64)
HR (bpm)—Standing		82.15 (11.64)
SDNN (ms)—Standing		43.64 (13.65)
RMSSD (ms)—Standing		33.40 (12.21)
pNN50 (%)—Standing		13.10 (12.63)
LF (Hz)—Standing		0.06163 (0.027)
HF (Hz)—Standing		0.05899 (0.028)
LF/HF—Standing		1.14254 (0.471)
**Characteristics**	**Answers**	**Frequency (Percentage)**
Sex ^b^	Female	30 (85.7)
	Male	5 (14.3)
Marital status ^b^	Unmarried	8 (22.9)
	Married	20 (57.1)
	Divorced	6 (17.1)
	Separated	1 (2.9)
Number of children ^b^	0	10 (28.6)
	1	2 (5.7)
	2	18 (51.4)
	3	3 (8.6)
	4	2 (5.7)
Body part with chronic pain ^b^	Neck	9 (25.7)
	Neck, lower back	5 (14.3)
	Neck, shoulder, upper extremities	4 (11.4)
Visit in doctor for neck pain ^b^	Yes	14 (40)
	No	21 (60)
Medicine for neck pain ^b^	Yes	11 (31.4)
	No	23 (68.6)
Physiotherapy treatment for neck pain ^b^	Never	22 (62.9)
	Rarely	7 (20)
	Often	6 (17.1)
Smoking ^b^	Yes	9 (25.7)
	No	26 (74.3)
Last time neck pain ^b^	Right now	25 (71.4)
	Today	4 (11.4)
	Last night	2 (5.7)
	2 months ago	1 (2.9)
	1 week ago	3 (8.6)

BMI, Body Mass Index; NRSr, Numerical Rating Scale at rest; NRSm, Numerical Rating Scale during active cervical spine movement; NDI, Neck Disability Index; HR, Heart Rate; SDNN, Standard Deviation of Normal-to-Normal RR intervals; RMSSD, Root Mean Square of the Successive Differences; pNN50, NN50 count (Number of pairs of adjacent NN intervals differing by more than 50 ms in the entire recording) divided by the total number of all NN intervals; LF, Low Frequency; HF, High Frequency; LF/HF, Low Frequency to High Frequency Ratio. ^a^ Values presented as mean (standard deviation). ^b^ Values presented as absolute number (percentage).

**Table 2:** Correlation r between Heart Rate Variability (HRV) indices, pain variables and range of cervical motion with Pearson’s correlation coefficient.

Variables	NRSm	NDI	PCS	RM	H	EQ-VAS	F	E	LR
NRSm	1								
NDI	0.545 **	1							
PCS	0.605 **	0.544 **	1						
RM	0.553 **	0.463 **	0.893 **	1					
H	0.514 **	0.588 **	0.907 **	0.672 **	1				
EQ-VAS	−0.347 *	−0.334 *	−0.402 *	−0.334	−0.466 **	1			
F	−0.079	−0.107	−0.230	−0.132	−0.239	0.185	1		
E	0.117	−0.023	0.055	0.075	0.003	0.005	0.284	1	
LR	−0.263	−0.437 **	−0.347 *	−0.346 *	−0.319	0.394 *	0.299	0.386 *	1
**Supine**									
HRV	−0.100	−0.198	−0.215	−0.173	−0.196	0.113	0.213	−0.131	0.008
HR (bpm)	0.022	0.072	0.293	0.293	0.217	−0.123	−0.021	0.098	−0.081
SDNN (ms)	−0.010	−0.088	−0.192	−0.129	−0.231	0.162	0.128	−0.097	0.137
RMSSD (ms)	−0.142	−0.217	−0.226	−0.185	−0.200	0.039	0.239	−0.170	−0.002
LF (Hz)	0.000	0.088	−0.122	−0.084	−0.158	0.083	−0.046	−0.141	−0.081
LF/HF	0.059	0.298	−0.035	0.052	−0.091	0.116	−0.244	−0.174	−0.267
**Sitting**									
HRV	−0.308	−0.285	−0.185	−0.149	−0.194	0.208	0.126	−0.012	0.191
HR (bpm)	−0.014	0.050	0.284	0.194	0.316	−0.141	0.024	0.206	0.004
SDNN (ms)	−0.162	−0.128	−0.275	−0.231	−0.232	0.127	0.026	−0.058	−0.035
LF/HF	−0.156	−0.041	−0.268	−0.188	−0.327	−0.087	−0.054	0.070	−0.132
**Standing**									
HRV	−0.230	−0.408 *	−0.321	−0.187	−0.370 *	0.222	0.056	−0.005	0.346 *
HR (bpm)	−0.062	0.033	0.301	0.214	0.327	−0.241	−0.149	0.056	−0.114
RMSSD (ms)	−0.217	−0.385 *	−0.321	−0.190	−0.371 *	0.213	0.081	−0.027	0.324
LF (Hz)	0.011	0.039	−0.044	−0.013	−0.115	0.038	−0.080	−0.038	−0.123
HF (Hz)	−0.275	−0.117	−0.226	−0.143	−0.241	0.212	0.236	0.020	0.268

HR, Heart Rate; SDNN, Standard Deviation of Normal-to-Normal RR intervals; RMSSD, Root Mean Square of the Successive Differences; LF, Low Frequency; HF, High Frequency; LF/HF, Low Frequency to High Frequency Ratio; NRSm, Numerical Rating Scale during active cervical spine movement; NDI, Neck Disability Index; PCS, Pain Catastrophizing Scale; RM, Rumination; H, Helplessness; EQ-VAS, Visual Analogue Scale of EQ-5D-5L; F, cervical Flexion; E, cervical Extension; LR, cervical Left Rotation. * *p* < 0.05; ** *p* < 0.01.

**Table 3:** Correlation r between Heart Rate Variability (HRV) indices, pain variables, and range of cervical motion with Spearman’s correlation coefficient.

Variables	Chronicity	NRSr	MG	EuroQol	RLF	LLF	RR
Chronicity	1						
NRSr	0.549 **	1					
MG	0.384 *	0.099	1				
EuroQol	−0.146	−0.195	−0.390 *	1			
RLF	−0.133	−0.118	−0.124	0.028	1		
LLF	−0.212	−0.078	−0.054	0.154	0.603 **	1	
RR	−0.077	−0.179	−0.234	0.516 **	−0.124	0.003	1
**Supine**							
pNN50 (%)	−0.152	0.176	−0.310	0.162	−0.129	−0.243	0.008
HF (Hz)	−0.169	0.191	−0.140	−0.024	−0.059	−0.071	−0.039
**Sitting**							
RMSSD (ms)	−0.116	−0.098	−0.130	0.199	−0.142	−0.045	0.190
pNN50 (%)	−0.060	−0.030	−0.149	0.190	−0.084	0.000	0.174
LF (Hz)	−0.244	−0.097	−0.014	0.200	0.146	0.062	0.103
HF (Hz)	−270	−0.061	−0.042	0.266	−0.042	−0.028	0.124
**Standing**							
SDNN (ms)	−0.181	0.160	−0.278	0.214	−0.090	−0.002	0.132
pNN50 (%)	−0.174	−0.214	−0.252	0.384 *	0.173	0.176	0.450 **
LF/HF	0.141	0.055	0.324	−0.393 *	−0.041	0.110	−0.352 *

pNN50, NN50 count (Number of pairs of adjacent NN intervals differing by more than 50 ms in the entire recording) divided by the total number of all NN intervals; HF, High Frequency; RMSSD, Root Mean Square of the Successive Differences; SDNN, Standard Deviation of Normal-to-Normal RR intervals; LF, Low Frequency; LF/HF, Low Frequency to High Frequency Ratio; NRSr, Numerical Rating Scale at rest; MG, Magnification; EuroQol, descriptive system of EQ-5D-5L; RLF, Right cervical Lateral Flexion; LLF, Left cervical Lateral Flexion; RR, Right cervical Rotation. * *p* < 0.05; ** *p* < 0.01.

## Data Availability

The dataset is available upon request from the corresponding author.
